# Supplementary material for: An open-source framework for large-scale, flexible evaluation of biomedical text mining systems
Source: J Biomed Discov Collab. 2008 Jan 29;3:1. doi: 10.1186/1747-5333-3-1 (PMC2276192; doi:10.1186/1747-5333-3-1)
Supplement: Additional file 1 — Evaluation platform source code. The additional file contains the software evaluation framework discussed in this paper. The most current release can be downloaded from SourceForge.net [1]. See the accompanying README file for instructions on installation and use. Also included in the distribution is a collection of UIMA wrappers for some commonly used BioNLP tools and annotated corpora. [file 1747-5333-3-1-S1.zip › bionlp_uima_repository-1.0.1/docs/api/src-html/edu/uchsc/ccp/util/nlp/parser/corpus/GeniaCorpusParser.html]

```
001    /*
002     * GeniaCorpusParser
003     * 
004     *  This class implements a parser for the GENIA corpus, specifically, for the GENIA corpus XML file. The parser was develped around version 3.02 of the GENIA corpus.
005     *  It utilizes the LingPipe GeniaEntityChunkParser to do that actual parsing. 
006     *  Currently, this parser can return 
007     *     1) the XML text for a GENIA document; 
008     *     2) a String containing the document ID for the document; 
009     *     3) a list of String containing the sentences for the document; and
010     *     4) a mapping between the sentences and lists of entities for each sentence. 
011     *           The entities take the form of Strings using the format "spanStart-spanEnd: classMentionName"
012     *           
013     *  Created: 1/2/07
014     *  
015     */
016    
017    package edu.uchsc.ccp.util.nlp.parser.corpus;
018    
019    import java.io.BufferedReader;
020    import java.io.FileNotFoundException;
021    import java.io.FileReader;
022    import java.io.IOException;
023    import java.util.ArrayList;
024    import java.util.HashSet;
025    import java.util.List;
026    import java.util.Map;
027    import java.util.Set;
028    import java.util.regex.Matcher;
029    import java.util.regex.Pattern;
030    
031    import org.xml.sax.SAXException;
032    
033    import edu.uchsc.ccp.util.external.lingpipe.LingPipe_Util;
034    import edu.uchsc.ccp.util.nlp.annotation.AnnotationSet;
035    import edu.uchsc.ccp.util.nlp.annotation.Annotator;
036    import edu.uchsc.ccp.util.nlp.annotation.TextAnnotation;
037    import edu.uchsc.ccp.util.nlp.document.GenericDocument;
038    import edu.uchsc.ccp.util.nlp.mention.ClassMentionTypes;
039    
040    /**
041     * A simple parser for the GENIA corpus which utilizes the LingPipe utilities in order to extract entities and sentences from the corpus.
042     * 
043     * @author wab
044     * 
045     */
046    public class GeniaCorpusParser extends CorpusParser {
047    
048        public static final int DOCUMENT_COLLECTION_ID = 4;
049    
050        private final static String GENIA_ARTICLE_START_TAG = "<article>";
051    
052        private final static String GENIA_ARTICLE_END_TAG = "</article>";
053    
054        private final static String GENIA_DOCUMENT_ID_START_TAG = "<bibliomisc>";
055    
056        private final static String GENIA_DOCUMENT_ID_END_TAG = "</bibliomisc>";
057    
058        private final static String GENIA_TITLE_START_TAG = "<title>";
059    
060        private final static String GENIA_TITLE_END_TAG = "</title>";
061    
062        private final static String GENIA_ABSTRACT_START_TAG = "<abstract>";
063    
064        private final static String GENIA_ABSTRACT_END_TAG = "</abstract>";
065    
066        private BufferedReader geniaFileReader;
067    
068        private String nextGeniaDocument = null;
069    
070        private String nextGeniaDocumentID = null;
071    
072        private List<String> nextGeniaDocumentSentences = null;
073    
074        private Map<String, List<String>> nextGeniaDocumentSentence2EntitiesMap = null;
075    
076        private static int annotationID = 0;
077    
078        private GenericDocument nextTitleDocument = null;
079    
080        private GenericDocument nextAbstractDocument = null;
081    
082        private boolean returnedTitleDocument = false;
083    
084        private boolean returnedAbstractDocument = false;
085    
086        private final Annotator annotator = new Annotator(8, "", "GENIA", "TSUJII");
087    
088        private final AnnotationSet annotationSet = new AnnotationSet(4, "GENIA Gold Annotations",
089                "Annotations extracted from the GENIA corpus.");
090    
091        public GeniaCorpusParser(String geniaXMLFile) {
092    
093            try {
094                geniaFileReader = new BufferedReader(new FileReader(geniaXMLFile));
095            } catch (FileNotFoundException e) {
096                error("Genia XML File not found. Please check the path. (" + geniaXMLFile + ")");
097                e.printStackTrace();
098                System.exit(-1);
099            }
100    
101        }
102    
103        @Override
104        public boolean hasNext() {
105            /* if we have not yet returned the abstract document, then return true */
106            if (returnedTitleDocument && !returnedAbstractDocument) {
107                return true;
108            } else {
109                /* we have already returned the abstract document, so check to see if there are new title and abstract documents to return */
110                try {
111                    return hasNextDocument();
112                } catch (IOException e) {
113                    error("Error while parsing the Bio1 corpus file.");
114                    e.printStackTrace();
115                    return false;
116                }
117            }
118        }
119    
120        @Override
121        public GenericDocument next() {
122            if (!returnedTitleDocument) {
123                returnedTitleDocument = true;
124                return nextTitleDocument;
125            } else {
126                returnedAbstractDocument = true;
127                return nextAbstractDocument;
128            }
129        }
130    
131        /*
132         * This method checks for the existence of the next document in the GENIA XML file by incrementing through the geniaFileReader(BufferedReader). If
133         * a document is found, it is stored in the nextGeniaDocument variable and true is returned. If no document is found, the nextGeniaDocument
134         * variable is set to null and false is returned.
135         */
136        private boolean hasNextDocument() throws IOException {
137            returnedTitleDocument = false;
138            returnedAbstractDocument = false;
139            nextGeniaDocument = null;
140            boolean hasNext = false;
141            Matcher m;
142            Pattern idPattern = Pattern.compile(GENIA_DOCUMENT_ID_START_TAG + "MEDLINE:(\\d+)" + GENIA_DOCUMENT_ID_END_TAG);
143            String line;
144            while ((line = geniaFileReader.readLine()) != null && !line.toLowerCase().contains(GENIA_ARTICLE_END_TAG)) {
145                /* iterate of lines in the genia XML file, look for the article start tag */
146                if (line.toLowerCase().contains(GENIA_ARTICLE_START_TAG)) {
147                    nextGeniaDocument = "";
148                    hasNext = true;
149                }
150                if (hasNext) {
151                    /* check the line for the document ID */
152                    if (line.trim().contains(GENIA_DOCUMENT_ID_START_TAG)) {
153                        m = idPattern.matcher(line);
154                        if (m.find()) {
155                            nextGeniaDocumentID = m.group(1);
156                        } else {
157                            error("DocumentID expected but not found.");
158                        }
159                    }
160    
161                    /* add the line to the nextGeniaDocument */
162                    nextGeniaDocument += (line + "\n");
163                }
164    
165            }
166    
167            /* add the article closing tag to the document (if there is one) */
168            if (hasNext) {
169                nextGeniaDocument += line;
170            
171    
172            /* extract the title and abstract from the GENIA document */
173            Pattern titlePattern = Pattern.compile(GENIA_TITLE_START_TAG + ".*?" + GENIA_TITLE_END_TAG, Pattern.MULTILINE);
174            m = titlePattern.matcher(nextGeniaDocument.replaceAll("\\n", " "));
175            String taggedTitleText = "";
176            if (m.find()) {
177                taggedTitleText = m.group();
178            } else {
179                error("Did not find a title.");
180            }
181    
182            Pattern abstractPattern = Pattern.compile(GENIA_ABSTRACT_START_TAG + ".*?" + GENIA_ABSTRACT_END_TAG, Pattern.MULTILINE);
183            m = abstractPattern.matcher(nextGeniaDocument.replaceAll("\\n", " "));
184            String taggedAbstractText = "";
185            if (m.find()) {
186                taggedAbstractText = m.group();
187            } else {
188                error("Did not find an abstract.");
189            }
190    
191            /* parse out the sentences and entities */
192            try {
193                LingPipe_Util.parseGeniaText("<?xml version=\"1.0\" encoding=\"UTF-8\"?>\n<set>\n" + taggedTitleText + "\n</set>");
194                List<String> titleSentences = LingPipe_Util.getGeniaSentencesFromParse();
195                Map<String, List<String>> titleSentence2EntitiesMap = LingPipe_Util.getGeniaEntitiesFromParse();
196    
197                int offset = 0;
198                String untaggedTitleText = "";
199                List<TextAnnotation> titleAnnotations = new ArrayList<TextAnnotation>();
200                for (String titleSent : titleSentences) {
201                    untaggedTitleText += (titleSent + " ");
202                    titleAnnotations.addAll(createEntityAnnotations(untaggedTitleText, titleSentence2EntitiesMap.get(titleSent), offset));
203                    titleAnnotations.add(createTextAnnotation(nextGeniaDocumentID, DOCUMENT_COLLECTION_ID, offset, titleSent.length()+offset, ClassMentionTypes.SENTENCE, titleSent, annotator, annotationSet, annotationID++));
204                    offset = untaggedTitleText.length();
205                }
206                untaggedTitleText = untaggedTitleText.trim();
207    
208                nextTitleDocument = new GenericDocument();
209                nextTitleDocument.setDocumentID(nextGeniaDocumentID + "-t");
210                nextTitleDocument.setDocumentText(untaggedTitleText);
211                nextTitleDocument.setAnnotations(titleAnnotations);
212    
213                LingPipe_Util.parseGeniaText("<?xml version=\"1.0\" encoding=\"UTF-8\"?>\n<set>\n" + taggedAbstractText + "\n</set>");
214                List<String> abstractSentences = LingPipe_Util.getGeniaSentencesFromParse();
215                Map<String, List<String>> abstractSentence2EntitiesMap = LingPipe_Util.getGeniaEntitiesFromParse();
216    
217                offset = 0;
218                String untaggedAbstractText = "";
219                List<TextAnnotation> abstractAnnotations = new ArrayList<TextAnnotation>();
220                for (String abstractSent : abstractSentences) {
221                    untaggedAbstractText += (abstractSent + " ");
222                    abstractAnnotations.addAll(createEntityAnnotations(untaggedAbstractText, abstractSentence2EntitiesMap.get(abstractSent),
223                            offset));
224                    abstractAnnotations.add(createTextAnnotation(nextGeniaDocumentID, DOCUMENT_COLLECTION_ID, offset, abstractSent.length()+offset, ClassMentionTypes.SENTENCE, abstractSent, annotator, annotationSet, annotationID++));
225                    offset = untaggedAbstractText.length();
226                }
227                untaggedAbstractText = untaggedAbstractText.trim();
228    
229                nextAbstractDocument = new GenericDocument();
230                nextAbstractDocument.setDocumentID(nextGeniaDocumentID + "-a");
231                nextAbstractDocument.setDocumentText(untaggedAbstractText);
232                nextAbstractDocument.setAnnotations(abstractAnnotations);
233    
234            } catch (SAXException e) {
235                e.printStackTrace();
236            }
237            }
238            // nextGeniaDocumentSentences = LingPipe_Util.getGeniaSentencesFromParse();
239            // nextGeniaDocumentSentence2EntitiesMap = LingPipe_Util.getGeniaEntitiesFromParse();
240    
241            return hasNext;
242        }
243    
244        /**
245         * converts an entity list, which looks like: "55-59:protein_molecule" into text annotations
246         * 
247         * @param entityList
248         * @param offset
249         * @return
250         */
251        private List<TextAnnotation> createEntityAnnotations(String documentText, List<String> entityList, int offset) {
252            annotationID = 0;
253            List<TextAnnotation> entityAnnotations = new ArrayList<TextAnnotation>();
254            Pattern entityPattern = Pattern.compile("(\\d+)-(\\d+):(.*)");
255            Matcher m;
256            for (String entity : entityList) {
257                System.out.println("EntityList: " + entity);
258                m = entityPattern.matcher(entity);
259                if (m.find()) {
260                    int spanStart = Integer.parseInt(m.group(1)) + offset;
261                    int spanEnd = Integer.parseInt(m.group(2)) + offset;
262                    String mentionType = m.group(3);
263                    entityAnnotations.add(createTextAnnotation(nextGeniaDocumentID, DOCUMENT_COLLECTION_ID, spanStart, spanEnd, mentionType,
264                            documentText.substring(spanStart, spanEnd), annotator, annotationSet, annotationID++));
265                } else {
266                    error("Invalid entity format: " + entity);
267                }
268            }
269            return entityAnnotations;
270        }
271    
272        /**
273         * Returns a list of sentences from the document that has just been parsed.
274         * 
275         * @return
276         */
277        public List<String> getNextDocumentSentences() {
278            return nextGeniaDocumentSentences;
279        }
280    
281        /**
282         * Returns a mapping from sentences to a list of entities. <br>
283         * The entities are represented as Strings using the following format: "spanStart-spanEnd: classMentionName"
284         * 
285         * @return
286         */
287        public Map<String, List<String>> getNextDocumentSentences2EntitiesMap() {
288            return nextGeniaDocumentSentence2EntitiesMap;
289        }
290    
291        /**
292         * Returns the document ID of the document that has just been parsed
293         * 
294         * @return
295         */
296        public String getNextDocumentID() {
297            return nextGeniaDocumentID;
298        }
299    
300        public static void outputAllMedlineIDs(String geniaFilename) {
301            Set<String> medlineIDs = new HashSet<String>();
302    
303            GeniaCorpusParser gcp = new GeniaCorpusParser(geniaFilename);
304    
305            while (gcp.hasNext()) {
306                String documentID = gcp.nextGeniaDocumentID;
307                if (!medlineIDs.contains(documentID)) {
308                    medlineIDs.add(documentID);
309                } else {
310                    System.err.println("Duplicate document ID found: " + documentID);
311                }
312            }
313    
314            System.out.println("There are " + medlineIDs.size() + " ids in the Genia collection.");
315            for (String id : medlineIDs) {
316                System.out.println(id);
317            }
318    
319        }
320    
321        //    
322        // /**
323        // * args[0] = the genia xml file <br>
324        // *
325        // * @param args
326        // */
327        // public static void main(String[] args) {
328        //        
329        // GeniaCorpusParser gcp = new GeniaCorpusParser(args[0]);
330        //
331        // // try {
332        // /* parse and output results for the first document in the genia xml file */
333        // if (gcp.hasNext()) {
334        // String documentText = gcp.next();
335        // System.out.println("DOCUMENT ID: " + gcp.nextGeniaDocumentID);
336        // System.out.println("DOCUMENT TEXT: " + documentText);
337        // List<String> documentSentences = gcp.getNextDocumentSentences();
338        // Map<String, List<String>> documentSentences2EntitiesMap = gcp.getNextDocumentSentences2EntitiesMap();
339        // for (String sentence : documentSentences) {
340        // System.out.println("DOCUMENT SENTENCE: " + sentence);
341        // System.out.println("SENTENCE ENTITIES: " + documentSentences2EntitiesMap.get(sentence));
342        // }
343        // }
344        // System.out.println("--------------------------------------------------------------------");
345        // /* parse and output results for the second document in the genia xml file */
346        // if (gcp.hasNext()) {
347        // String documentText = gcp.next();
348        // System.out.println("DOCUMENT ID: " + gcp.nextGeniaDocumentID);
349        // System.out.println("DOCUMENT TEXT: " + documentText);
350        // List<String> documentSentences = gcp.getNextDocumentSentences();
351        // Map<String, List<String>> documentSentences2EntitiesMap = gcp.getNextDocumentSentences2EntitiesMap();
352        // for (String sentence : documentSentences) {
353        // System.out.println("DOCUMENT SENTENCE: " + sentence);
354        // System.out.println("SENTENCE ENTITIES: " + documentSentences2EntitiesMap.get(sentence));
355        // }
356        // }
357        //
358        // /* cycle through the rest of the xml file */
359        // while (gcp.hasNext()) {
360        // String documentText = gcp.next();
361        // if (documentText == null) {
362        // System.err.println("Found null document text.");
363        // }
364        // }
365        //
366        //            
367        // GeniaCorpusParser.outputAllMedlineIDs(args[0]);
368        //            
369        // // } catch (IOException e) {
370        // // e.printStackTrace();
371        // // }
372        //
373        // }
374    
375        /**
376         * The remove operation is not currently supported.
377         */
378        public void remove() throws UnsupportedOperationException {
379            throw new UnsupportedOperationException();
380        }
381    
382        @Override
383        public void close() throws IOException {
384            geniaFileReader.close();
385        }
386    
387    }
```
